# 

**Published:** 1862-05

**Authors:** 


### CONTENTS.

### ORIGINAL CONTRIBUTIONS.

ART. XIII. Upon the Pathology of Milksickness. A Thesis, by Geo. Wheeler Jones, M.D. .... 267
ART. XIV. Retention of the Urine, caused by a Calculus in the Urethra, with remarks as to some of the more prominent causes of vesical retention in the young. By Swayne Wickersham, M.D. 284
ART. XV. Report upon the Sanitary Condition of the West Division of Chicago, By H. Webster Jones, M.D. .... 290

### ARMY CORRESPONDENCE.

Letter from H. Wardner, Surgeon, 12th Ill. Vols., .... 293
Letter from O. B. Ormsby, Asst.-Surgeon, 18th Regt. Ill. Vols., .... 299
Extract from a Letter from Dr. C. Dumreicher, .... 301

### SELECTIONS.

Lectures on New Remedies and their Therapeutical Applications. By Samuel R. Percy, M.D. .... 302

### BOOK NOTICES.

The Principles and Practice of Obstetrics. By Gunning S. Bedford, A.M., M.D., etc. .... 316
Minor Surgery. By F. W. Sargent, M.D., etc. .... 317
Nineteenth Annual Report of the Managers of the New York State Lunatic Asylum .... 317
Forty-fifth Annual Report, on the state of the Asylum for the relief of persons deprived of the Use of their Reason. Philadelphia .... 317
Annual Report of the Officers of the Indiana Hospital for the Insane. By James S. Athon, M.D., Superintendent. .... 318
The Action of the Voluntary Muscles. By Louis Mackall, M.D. .... 318

### EDITORIAL.

Case of Cerebral Congestion. By O. B. Ormsby, Asst.-Surgeon, 18th Regt. Ill. Vols. .... 319
Uterine Hemorrhage—H. Wanzer, M.D. .... 328
New York Ophthalmic Hospital, .... 328
General Literature and Art—Eclectic Magazine, .... 329
"Illinois Farmer," and "Prairie Farmer," .... 329

